# Anti-Lubricin Monoclonal Antibodies Created Using Lubricin-Knockout Mice Immunodetect Lubricin in Several Species and in Patients with Healthy and Diseased Joints

**DOI:** 10.1371/journal.pone.0116237

**Published:** 2015-02-02

**Authors:** Minrong Ai, Yajun Cui, Man-Sun Sy, David M. Lee, Ling Xiu Zhang, Katherine M. Larson, Kyle C. Kurek, Gregory D. Jay, Matthew L. Warman

**Affiliations:** 1 Howard Hughes Medical Institute and Department of Genetics, Case Western Reserve University, School of Medicine, Cleveland, OH, United States of America; 2 Department of Pathology, Case Western Reserve University, School of Medicine, Cleveland, OH, United States of America; 3 Division of Rheumatology, Immunology and Allergy, Brigham and Women’s Hospital, Boston, MA, United States of America; 4 Department of Emergency Medicine, Rhode Island Hospital, Providence, RI, United States of America; 5 School of Engineering, Brown University, Providence, RI, United States of America; 6 Department of Pathology, Boston Children’s Hospital, Boston, MA, United States of America; 7 Howard Hughes Medical Institute and Orthopaedics Research Laboratories, Boston Children’s Hospital, and Departments of Genetics and Orthopaedic Surgery, Harvard Medical School, Boston, MA, United States of America

## Abstract

Lubricin, encoded by the gene *PRG4*, is the principal lubricant in articulating joints. We immunized mice genetically deficient for lubricin (*Prg4*-/-) with purified human lubricin, and generated several mAbs. We determined each mAb’s binding epitope, sensitivity, and specificity using biologic samples and recombinant lubricin sub-domains, and we also developed a competition ELISA assay to measure lubricin in synovial fluid and blood. We found the mAbs all recognized epitopes containing O-linked oligosaccharides conjugated to the peptide motif KEPAPTTT. By western blot, the mAbs detected lubricin in 1 μl of synovial fluid from several animal species, including human. The mAbs were specific for lubricin since they did not cross-react with other synovial fluid constituents from patients with camptodactyly-arthropathy-coxa vara-pericarditis syndrome (CACP), who genetically lack this protein. The competition ELISA detected lubricin in blood samples from healthy individuals but not from patients with CACP, indicating blood can be used in a diagnostic test for patients suspected of having CACP. Lubricin epitopes in blood do not represent degradation fragments from synovial fluid. Therefore, although blood lubricin levels did not differentiate patients with inflammatory joint disease from healthy controls, epitope-specific anti-lubricin mAbs could be useful for monitoring disease activity in synovial fluid.

## Introduction

Lubricin is a large (>250kDa), secreted, highly glycosylated mucinous protein that is abundant in the synovial fluid of mammalian diarthrodal joints [1]. Lubricin is secreted by synovial fibroblasts [2] and superficial zone chondrocytes [3] as a monomer, dimer, or tetramer [4–6], and is responsible for boundary lubrication [7–9] and the protection of articular cartilage from friction-induced damage [10]. Lubricin is also present on the surface of tendons and tendon sheaths [11] and in the pericardial and pleural cavities, where it likely serves an anti-adhesive function. Other tissues including liver, kidney, and skeletal muscle also express lubricin mRNA, although the protein’s biologic role(s) in these tissues is unknown. In humans, genetic absence of lubricin causes the camptodactyly-arthropathy-coxa vara-pericarditis syndrome (CACP) [12,13]. Among the clinical consequences of CACP are non-inflammatory synovial cell hyperplasia, precocious joint failure, adhesions between tendons and tendon sheaths, and restrictive pericarditis [12]. Mice that have been genetically engineered to lack lubricin (*Prg4* ^-/-^) developed camptodactyly, synovial hyperplasia, and cartilage deterioration, similar to humans with CACP [14–16]. Acquired deficiency of lubricin, due to enhanced degradation following injury or inflammatory disease, may contribute to the deterioration of the involved joint [17]; *in vitro* studies have shown that reducing lubricin between loaded and sliding cartilage explants leads to increases in the coefficient of friction and in chondrocyte apoptosis [18].

Full-length human lubricin is a 1404 amino acid residue protein encoded by the gene *PRG4*. *PRG4* gene products have also been identified as superficial zone protein (SZP), megakaryocyte-stimulating factor (MSF) and hemangiopoietin (HAPO). The endogenous role for lubricin/SZP at the cartilage surface is likely the same as for lubricin in synovial fluid, while HAPO is a putative growth factor in the maturation of bone marrow hematopoietic cells [19]. In humans, more than half of the lubricin polypeptide contains 2 large mucin-like domains. The first mucin domain contains multiple copies of the octapeptide motif KEPAPTTT. The threonine residues in this motif are believed to be critical sites for O-linked sugar modifications where β(1–3)Gal-GalNAc is incompletely capped with NeuAc [20]. Limited branching of O-linked sugar chains has been observed in lubricin from patients with osteoarthritis (OA) and rheumatoid arthritis (RA) [21]. The second mucin-like domain is also poly-threonine rich but with a less recognizable motif structure. A hemopexin-like (PEX) domain at the carboxyl-terminus of the protein likely confers specificity for matrix binding [22]. Proteolytic cleavage within the PEX domain by subtilisin-like proprotein convertases has been observed [23].

Changes in lubricin abundance and intactness have been reported in animal joint injury models and in humans with OA and RA. For example, lubricin abundance in synovial fluid decreased after cruciate ligament injury in rabbits [24] and humans [25]. In a rabbit model of RA induced by methylated bovine serum albumin, decreased levels of synovial fluid lubricin were also observed, and these decreases were due to decreased expression and increased proteolysis [26]. Synovial fluid from patients with active RA and OA exhibited lubricin degradation [23]. Therefore, tools that permit sensitive and reproducible studies of lubricin quantity and quality may be useful for understanding the role of this protein in maintaining joint health and predisposing to joint failure.

In order to better understand how lubricin is synthesized, post-translationally modified, and degraded, we generated anti-human lubricin monoclonal antibodies (mAbs) in *Prg4* ^-/-^ mice. We tested the mAbs on synovial fluid and blood samples from patients with CACP to show the mAbs are sensitive and specific for lubricin. We also showed that the mAbs recognized lubricin from several other species. We delineated each mAb’s epitope and found that all mAbs recognized an epitope with the same polypeptide motif; however, each mAb exhibited different sensitivity to O-linked oligosaccharide post-translational modifications of this motif. These new mAbs could detect lubricin by immunoblotting, immunoprecipitation, immunohistology, and competition ELISA. Anti-lubricin mAbs with defined epitopes, sensitivities, and specificities will be useful in diagnosing patients suspected of having CACP and in studying lubricin in patients and animal models affected by joint disease.

## Materials and Methods

The University Hospitals of Cleveland, the Rhode Island Hospital, the Brigham and Women’s Hospital, and the Boston Children’s Hospital Institutional Review Boards approved this study. Written informed consent and assent (for minors older than 8 years-of-age) were obtained by Dr. Warman in the Division of Human Genetics at University Hospitals of Cleveland or the Department of Orthopaedic Surgery at Boston Children’s Hospital from participants with CACP, their unaffected siblings, and their carrier parents (n = 6). Written consent was not required, nor obtained, for fully anonymized samples that would otherwise have been discarded following a clinical procedure. Anonymized blood samples from adult patients with rheumatoid arthritis and controls (n = 60) were provided by the Division of Rheumatology, Immunology and Allergy, Brigham and Women’s Hospital, Boston, MA. Anonymized adult synovial fluid samples were provided by the Department of Orthopaedic Surgery at University Hospitals of Cleveland, Cleveland OH and the Department of Emergency Medicine at Rhode Island Hospital, Providence RI (n = 6). Anonymized synovial tissue sections from patients with degenerative arthritis were obtained from the Department of Pathology, Boston Children’s Hospital, Boston, MA (n = 4). The Institutional Animal Care and Use Committees at Case Western Reserve University, Cleveland, OH and Brown University, Providence, RI also approved these studies. Cow, pig, and goat synovial fluid were obtained from a local abattoir. Dog, rat, and guinea pig synovial fluid were obtained after euthanasia by exsanguination under deep anesthesia. No animal was euthanized specifically for this study. Synovial fluid was collected from animals that had been euthanized under other IACUC approved protocols.

### Generation and purification of anti-lubricin monoclonal antibodies (mAbs)

Purified human lubricin was obtained as described [7]. *Prg4* ^-/-^ mice [14] were immunized with lubricin mixed with complete Freund adjuvant using standard methods. Following sacrifice, splenocytes were collected and hybridomas generated by fusion with SP2/0 cells. Hybridomas were cultured in HAT-RPMI media for two weeks after which direct ELISA was performed to test cell culture supernatants for the presence of anti-lubricin antibody. Positive hybridomas were injected into the abdominal cavity of nude mice primed with pristane. Ascites fluid containing high concentrations (1~2 mg/ml) of anti-lubricin antibody was collected. After centrifugation for 5 min to get rid of lipid and blood cell contamination, the ascites fluid was filtered with a 0.8 μm filter and stored in -20°C until use or purification. Antibody was purified using Protein G agarose beads.

Purified antibody was labeled with biotin using EZ-link Sulfo-NHS-LC-Biotin reagent (Pierce, Rockford, IL). The antibody (0.2 mg/ml) was mixed with Sulfo-NHS-biotin reagent (2 mM) at 4°C for 2 hr. Unreacted Sulfo-NHS-biotin was removed by dialysis in PBS buffer at 4°C overnight. The concentration of the biotin-labeled antibody was determined by OD readings at 280 nm wavelength.

### Collection of synovial fluid and blood samples

Human synovial fluid was obtained following diagnostic aspiration of affected knees. Synovial fluid samples were stored at -20°C. Plasma and/or serum samples were obtained from patients with CACP, OA, RA and healthy controls. Plasma from patients and unaffected relatives with CACP were stored at 4°C, while control and RA serum samples were stored at -20°C. Synovial fluid aspirates from different species were collect immediately following euthanasia and stored at -20°C.

### Recombinant Human Lubricin (rhPRG4)

Full-length rhPRG4, comprising 1404 amino acids (GenBank: NP_005798), was expressed by transformed Chinese hamster ovary cells (SBH Sciences, Natick, MA). This product appears as a monomer and homodimer on agarose gel electrophoresis and bears the same apparent molecular weight as human synoviocyte lubricin [5].

### Expression of lubricin sub-domains

cDNA from the human chondrosarcoma cell line SW1353 (ATCC, Manassas, VA) was used as template to PCR amplify lubricin (*PRG4*) coding sequence for sub-cloning into an expression vector that includes a signal peptide and a hemaglutinin (HA) epitope tag (YPYDVPDYA). Expression constructs included lubricin amino acid residues 26–278 (N), 281–857 (Mu1), 281–627 (Mu1a), 281–377 (Mu1b), 629–857 (Mu1c), 857–1140 (Mu2), and 1141–1404 (C). Because polypeptide Mu1b is small, human IgG-Fc coding sequence was added 3’ and in-frame with the Mu1b fragment (Mu1b-Fc). The Fc expression construct, encodes a signal peptide followed by an HA epitope tag and a human IgG-Fc domain using pcDNA3. To make the T-Fc expression construct, DNA encoding an octapeptide motif (KEPAPTTT) was introduced into the Fc expression construct between the HA epitope tag and the IgG-Fc domain. Variants of the T-Fc recombinant protein were generated by site-directed mutagenesis using a Quickchange kit (Stratagene, La Jolla, CA).

To express recombinant protein, HEK-293T cells were cultured in DMEM with 10% fetal bovine serum (FBS) at 37°C with 5% CO_2_, then transfected using serum free DMEM and Lipofectamine Plus (Invitrogen, Carlsbad, CA) according to manufacturer’s protocol. Serum-free conditioned medium containing recombinant proteins was collected 48 hr after transfection.

### Enzymatic deglycosylation of native and recombinant lubricin

Five μg of purified human lubricin was diluted in 100 μl reaction buffer (50 mM NaHPO_3_, pH 7.2, 0.01% w/v BSA) and treated with 80 mU of neuraminidase (ProZyme, Hayward, CA) and/or 4 mU of O-glycosidase (Roche, Germany) at 37°C overnight. The digests were mixed with 100 μl of 2X SDS-PAGE loading buffer containing 2% v/v BME and boiled for 5 min. Two μl were then electrophoresed in 4–20% gradient gels (Bio-Rad, Hercules, CA) and transferred to Immobilon P-PVDF (Millipore, Bedford, MA).

Twenty μl of serum-free conditioned medium from HEK-293T cells containing rhPRG4 were treated with 30 mU of neuraminidase and/or 2 mU of O-glycosidase in a 50 μl reaction at 37°C overnight. The digested samples were then mixed with 50 μl of 2X SDS-PAGE loading buffer and boiled for 5 min. Five μl of sample was electrophoresed and transferred to PVDF.

### Immunodetection of lubricin on Western blots

For synovial fluid samples: Following SDS-PAGE electrophoresis and transfer to Immobilon P-PVDF, the membranes were incubated in TBS (20mM Tris and 150mM NaCl, PH 7.5) containing 5% w/v non-fat dried milk for 2 hr at 4°C. Following 3 washes in TBST (TBS with 0.01% w/v Tween 20), one of the mAbs (1:5000 dilution in TBST) or a polyclonal antibody J108N [14] (1:2000 dilution in TBST) was incubated with the membrane for 16 to 20 hr at 4°C. Following 3 washes in TBST, the membranes were incubated with horseradish peroxidase (HRP) conjugated secondary antibodies (either goat anti-mouse IgG, goat anti-mouse IgG-Fab specific, goat anti-rabbit IgG, or HRP-streptavidin, Pierce, Rockford, IL) at 1:5000 dilution. Following 3 washes in TBST, immunoreactive bands were detected by chemiluminescence using the ECL Plus Western Blotting detection kit (GE Healthcare, Buckinghamshire, UK). The chemiluminescence signal was then captured with BioMax film (Kodak, Rochester, NY).

For purified mucin samples: Bovine submaxillary mucin and porcine gastric mucin (both from Sigma-Aldrich, St. Louis, MO) were dissolved in PBS. Following SDS-PAGE electrophoresis on a 4–12% polyacrylamide gels at 200 V for 1 hr and transferred at 24V to nitrocellulose for 1 hr, membranes were incubated in PBST (10 mM KH_2_PO_4_, 0.15 M NaCl, 30 mM Na_2_HPO_4_7H_2_O, pH 7.4, with 0.05% w/v Tween 20) containing 5% w/v non-fat dried milk for 1 hr at room temperature (RT), washed 3 times with PBST and incubated overnight at 4°C with of the mAbs in PBST containing 5% w/v bovine serum albumin (BSA). Membranes were then washed 3 times in PBST and incubated with secondary goat anti-mouse-IRdye-800 (0.1μl/ml) at RT for 1 hr. Immunoreactive bands were then detected via Odyssey Infrared Imager Scanner (Biosciences, LI-COR Inc.).

### Competition ELISA assay

Individual wells in polystyrene 96-well plates (Corning Incorporated, Corning, NY) were coated using 100 μl of purified human lubricin [7] in PBS (10 μg/ml) at 4°C overnight. Wells were then rinsed 3 times with PBS and blocked with 200 μl 5% (w/v) BSA in PBST at RT for 2 hr.

Serial dilutions of purified human lubricin (0–2 μg/ml) and purified rhPRG4 (0–100 μg/ml) and dilutions of human synovial fluid, human serum and plasma were made using PBS. For the assays reported in this study, 1:500 dilutions of synovial fluid and 1:50 dilutions of serum or plasma were used. Fifty μl of standard or 50 μl of sample were added to ELISA plate wells followed by 50 μl of biotin-conjugated mAb-7h12 (0.1 μg/ml in PBS) and incubated at RT for 1 hr. Wells were then rinsed 3 times with PBST and mAB-7h12 binding was detected by adding 100 μl of horseradish peroxidase conjugated Streptavidin (Pierce, Rockford, IL), diluted 1:1000 in PBS for 1 hr at RT, rinsing the wells 5 times with PBST, and developing with a horseradish peroxidase substrate kit (Bio-Rad, Hercules, CA) using 100 μl tetramethyl benzidine (TMB). A dark blue color was developed after 5–10 min, which was then stopped using 100 μl of 1N H_2_SO_4_. OD_415_ nm readings were obtained using a plate reader. A standard curve was obtained for each competition ELISA experiment. Lubricin concentrations in the test samples were determined by comparing the OD_415_ nm readings of the samples with that experiment’s standard curve. In the case of the rhPRG4 competition ELISA, which was documented by digital photography, the substrate was ABTS Solution (Life Technologies, Grand Island, NY) and was quantitated by OD_450_ nm.

### Immunoprecipitation of lubricin from synovial fluid and blood

Biotin labeled mAb-7h12 (4 μg) was mixed with an 80 μl slurry of Streptavidin agarose beads (Pierce, Rockford, IL) in a total of 1 ml PBS-T buffer (PBS with 0.1% Tween-20) and then agitated at RT for 30 min followed by 2 washes with the PBS-T buffer. Beads were then re-suspended in a total volume of 1 ml PBS-T buffer that included 100 μl plasma or serum, 5 μl synovial fluid, or 0.5 μg purified human or bovine lubricin) and again agitated for 1 hr at RT. After washing 5x with 1 ml PBS-T, the antibody-bound protein was eluted with 150 μl glycine-HCl (100 mM, pH 3.0) for 10 min. The supernatants were neutralized with 15 μl 1M Tris-HCl (pH 8.5), boiled with 2x SDS-PAGE loading buffer, and subjected to gel electrophoresis followed by Western blot analysis.

### Immunohistochemical detection of lubricin in human tissues

Six μm sections were prepared from formalin fixed paraffin embedded human synovial tissue samples from individuals without joint disease as well as from those with degenerative arthritis and CACP. Sections were deparaffinized in xylene, rehydrated, and antigen retrieval was performed in citrate buffer (pH 6) for 4 min at 125°C in a pressure cooker. Endogenous tissue peroxidase activity was quenched with 3% sodium hydroxide for 10 minutes and, following washes in PBS, nonspecific antibody binding was blocked with 1.5% blocking serum (Sigma-Aldrich). Antibody incubation and detection was performed using the VectaStain Elite Mouse IgG ABC kit (Vector Laboratories, Burlingame, CA) according to the manufacturer’s recommended protocol with appropriate controls. The antibody mAb-7h12 was used at a 1:400 dilution. Detection of the avidin/biotin/peroxidase-conjugated secondary antibody was performed using DAB FastTabs substrate (Sigma-Aldrich) for 5 minutes, followed by counterstaining with hematoxylin or hematoxylin and eosin. Slides were dehydrated and mounted for light microscopic analysis.

## Results

### Generation of anti-lubricin monoclonal antibodies

Using direct ELISA, we screened >100 hybridomas derived from lubricin immunized *Prg4* ^-/-^ mice for anti-lubricin antibodies. The use of *Prg4* ^*-/-*^ mice enabled us to generate mAbs that react with lubricin from multiple mammalian species, including human, cow, pig, goat, dog, and rat. Five hybridomas 7h12, 9g3, 5c11, 8e3 and 6a8 were recovered. The mAbs produced by these hybridomas were all IgG class.

**Fig 1:**
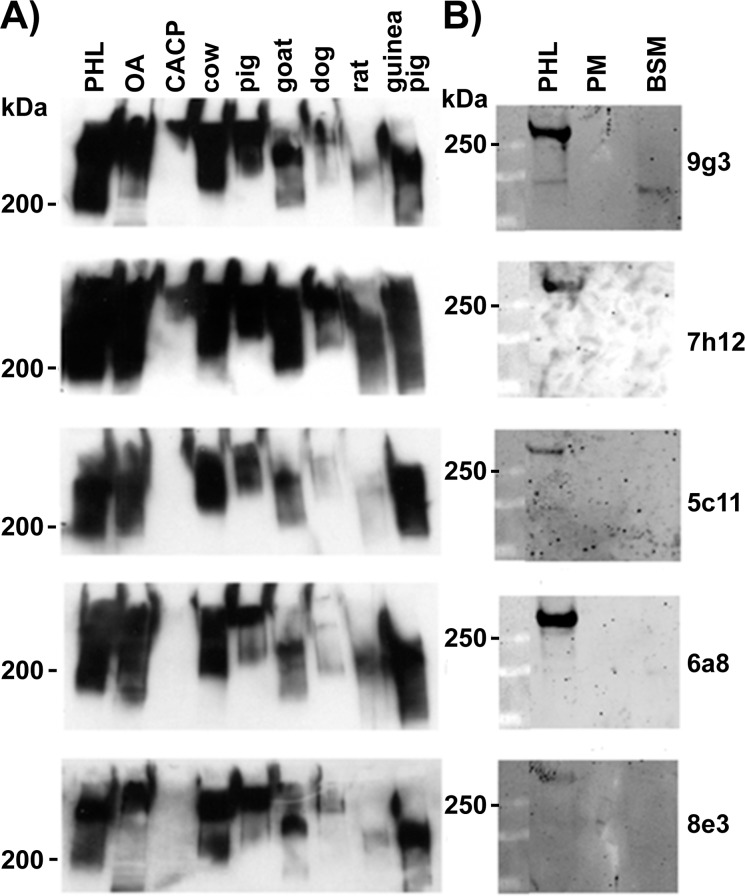
Western blots probed with anti-lubricin mouse monoclonal antibodies (mAbs) 9g3, 7h12, 5c11, 6a8, and 8e3 detect lubricin in synovial fluid but do not detect other mucins. **(A)** Five μg of human lubricin purified from synovial fluid (PHL), and 1 μl of synovial fluid recovered from patients with osteoarthritis (OA), CACP, or from bovine, porcine, goat, dog, rat and guinea pig were electrophoresed under reducing conditions on 4–20% SDS-PAGE gradient gels, transferred to PVDF, and immunodetected using the biotin labeled mAbs. Note that the mAbs detect protein in the synovial fluid from patients with OA and from other species, but not from the patient with CACP, who is genetically deficient for lubricin. **(B)** 0.7 μg of Purified human lubricin (PHL), porcine gastric mucin (PM) and bovine submaxillary mucin (BSM) were electrophoresed under non-reducing conditions on 4–12% SDS-PAGE gradient gels, transferred to nitrocellulose and immunodetected using the mAbs. For mAb-8e3, 1.4 μg of PHL was loaded. Note that anti-lubricin mAbs did not cross react with BSM or PM.

By Western blot, each mAb detected lubricin in 1 μl of synovial fluid from humans and several other mammalian species (Fig. 1A). The specificity for lubricin was demonstrated by the lack of immunoreactivity against synovial fluid from patients with CACP, who genetically lack this protein (Fig. 1A). The mAbs exhibited no cross reactivity to μg amounts of bovine and porcine mucins in Western blots (Fig. 1B).

### The mAbs recognize an O-linked oligosaccharide-containing octapeptide epitope

**Fig 2:**
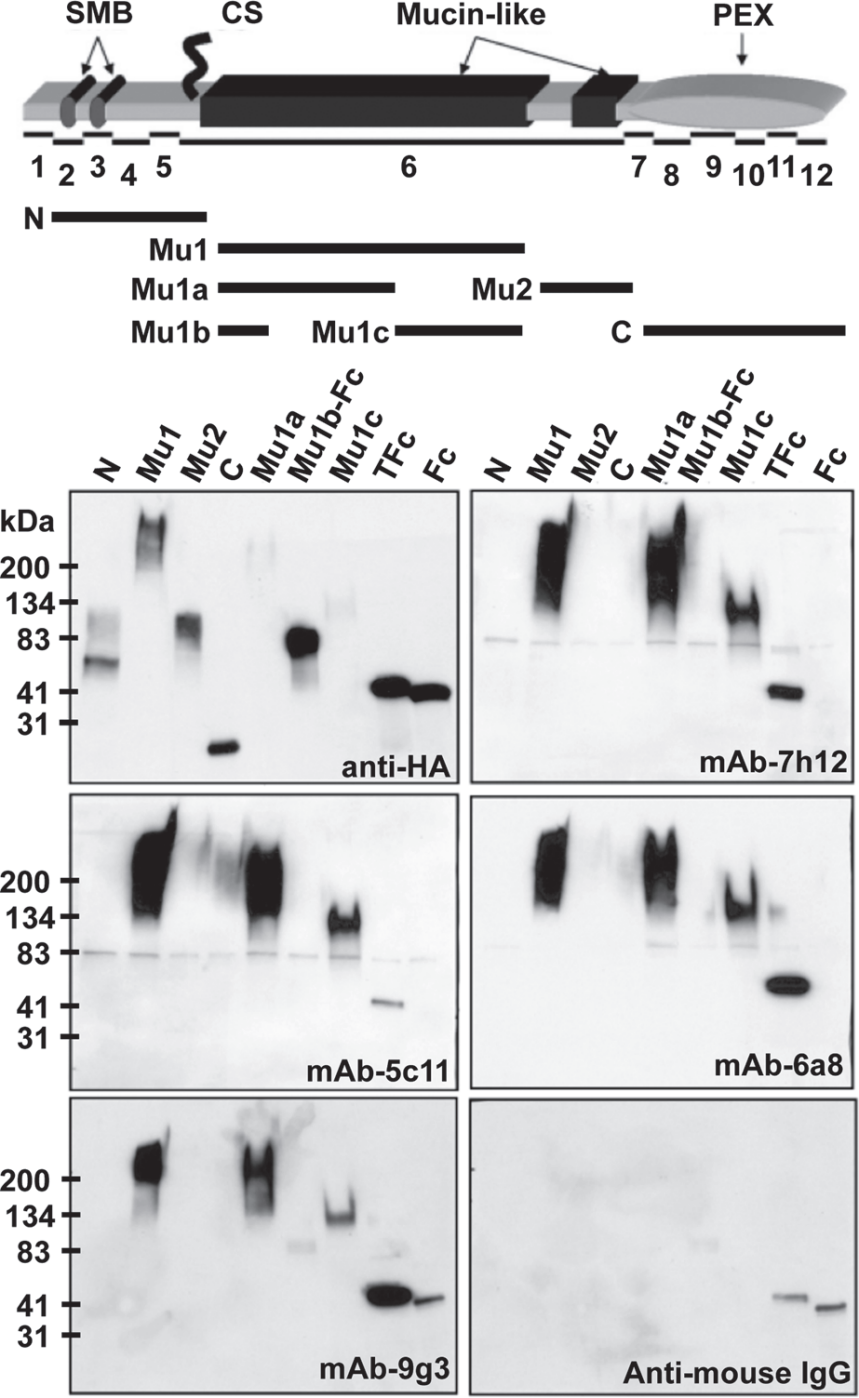
The mAbs detect an octapeptide motif present in the first mucin-like domain of human lubricin. Different domains of lubricin (depicted in the top panel beneath a schematic of the protein and its 12 coding exons) were cloned into a mammalian expression construct downstream of a signal peptide sequence and a Hemaglutinin epitope-tag (HA) sequence. Domain Mu1b was also fused to a human IgG-Fc fragment (Mu1b-Fc). An octapeptide motif (KEPAPTTT), which occurs multiple times within the first mucin-like domain (Mu1), was also fused to the IgG-Fc fragment (T-Fc). These constructs were transiently transfected into 293T cells. Serum-free conditioned media were collected and subjected to SDS-PAGE on 4–20% gradient gels. Anti-HA antibody detected all recombinant proteins in the media (HA), although weak immunodetectable bands occurred for Mu1a and Mu1c. Monoclonal antibodies 9g3, 7h12, 5c11 and 6a8 detected the secreted first mucin domain Mu1, Mu1a and Mu1c, but not Mu1b or the second mucin domain Mu2. Mu1b is the first 98 AA of the first mucin domain and does not contain KEPAPTTT; instead it has “KEPTPTT” and other “ETTT”-containing repeats. The only peptide motifs that Mu1a and Mu1c share is: “KEPAPTTP”. Antibodies 9g3, 7h12 and 6a8 also strongly recognized the KEPAPTTT containing recombinant protein (T-Fc), while 5c11 weakly interacted with T-Fc. Horseradish peroxidase (HRP) conjugated anti-mouse IgG, which was used as secondary antibody to detect 9g3, showed weak cross reactivity to human IgG-Fc. HRP-conjugated Streptavidin was used as a secondary antibody to detect biotin-labeled 7h12, 5c11 and 6a8, and showed no cross-reactivity by itself (data not shown).

**Fig 3:**
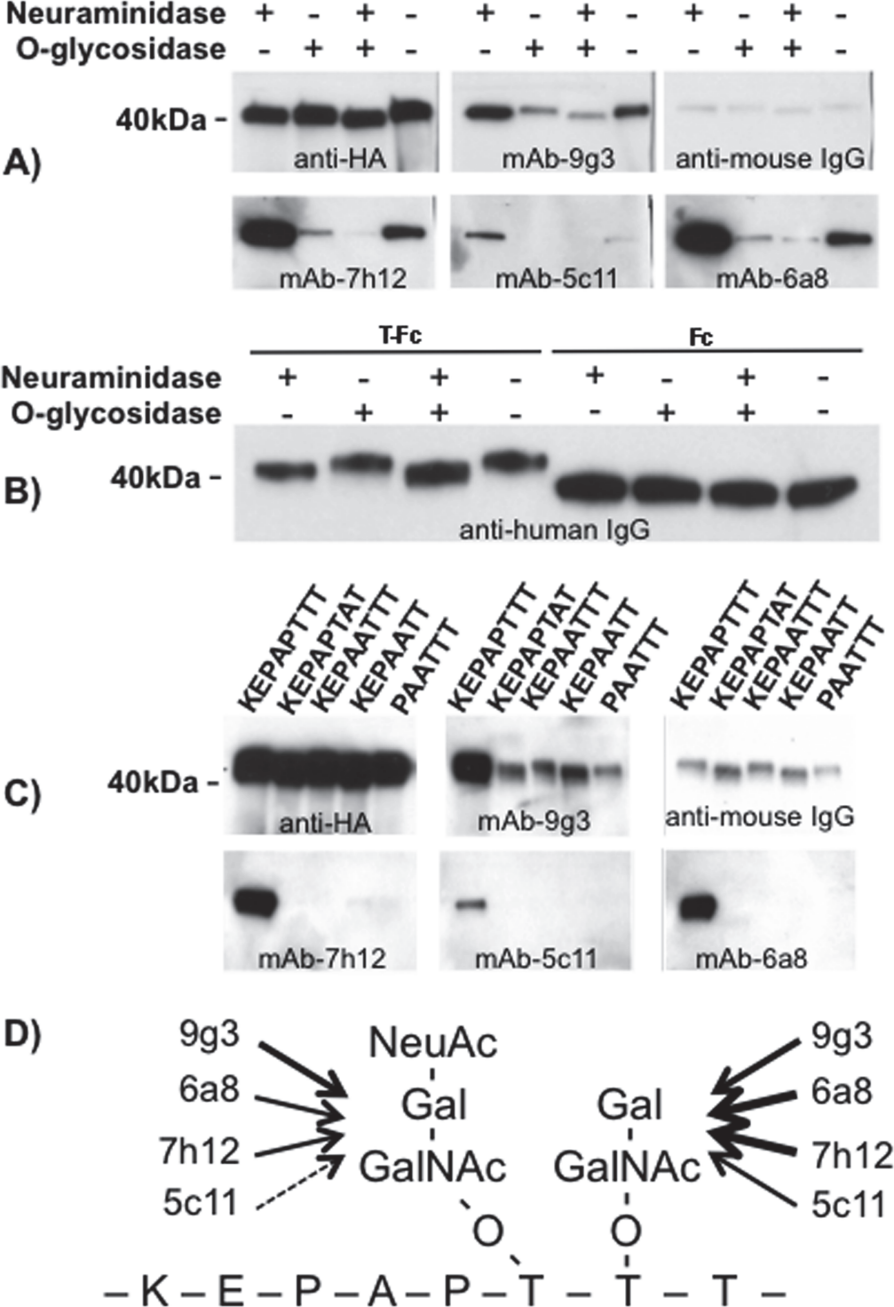
The mAbs recognize an epitope that contains an O-linked glycan modification of the octapeptide K-E/A-P-A-P-T-T-T/A/P. Serum-free conditioned media from 293T cells expressing T-Fc (i.e., KEPAPTTT) recombinant protein or variants of this octapeptide motif were resolved on reducing SDS-PAGE gel and immunodetected with the mAbs. **(A)** Recombinant T-Fc immunodetected using antibodies indicated in each panel. Removal of O-linked glycans by enzymatic digestion with neuraminidase and O-glycosidase caused loss or attenuated recognition by all mAbs, except anti-HA antibody. Interestingly, all mAbs showed significantly increased immunoreactivity to the recombinant protein treated with neuraminidase alone, suggesting sialic acid modifications that usually occur at sugar chain termini interfere with epitope-antibody interactions. **(B)** Recombinant HA-KEPAPTTT-Fc (T-Fc) and HA-IgG-Fc (Fc) proteins were treated by neuraminidase and/or O-glycosidase and immunodetected using anti-human IgG-Fc. Note sugar modification on the KEPAPTTT peptide, but not on the Fc fragment, is responsible for changes in polypeptide mobility upon deglycosylation. **(C)** Recombinant octapeptides fused downstream of HA and upstream of IgG-Fc immunodetected using antibodies indicated in each panel. Anti-HA antibody showed that all mutant recombinant proteins were expressed. Recombinant protein HA-KEPAPTTT-Fc (1) was immunodetected by all monoclonal antibodies. These antibodies did not detect the variant forms of the recombinant protein: HA-KEPAPTAT-Fc (2); HA-KEPAATTT-Fc (3); HA-KEPAPTT-Fc (4); HA-PAPTTT-FC (5). Note that the anti-mouse IgG, used as secondary antibody for mAb-9g3, showed strong cross-reactivity with the human IgG-Fc present in all recombinant proteins. HRP-conjugated Streptavidin was used as a secondary antibody to detect biotin-labeled mAbs 7h12, 5c11 and 6a8, and showed no background by itself (data not shown). **(D)** Schematic depicting the peptide and O-linked glycosylation motifs that occur commonly in the mucin-like domain of human lubricin. Sialylated and non-sialylated O-linked oligosaccharides can be added to the Threonine (T) residues; two potential oligosaccharides (sialylated and non-sialylated) are indicated and their relative affinities to the mAbs are indicated by the arrow weights.

To identify the epitope recognized by the mAbs we tested each antibody against subdomains of human lubricin (Fig. 2). The antibodies recognized the first mucin-like domain (Mu1). They did not detect the second mucin-like domain (Mu2), the amino-terminal globular domain (N), or the carboxyl-terminal PEX domain (C). Because Mu1 is enriched with an octapeptide motif (KEPAPTTT) that is present ~ 20 times in humans, we tested whether this peptide motif constitutes the epitope for the mAbs. We expressed a recombinant protein (T-Fc) that contains a single copy of this octapeptide motif in mammalian cells and confirmed that it contains the reactive epitope for all mAbs (Figs. 2 and 3A). Since the octapeptide motif is partially degenerate in human and other mammalian lubricins, we altered amino acid residues within the motif to fully define the epitope (Fig. 3B). We found that the consensus peptide motif recognized by these mAbs is K-E/A-P-A-P-T-T-T/A/P (Fig. 3B).

Threonine residues are sites of O-linked glycosylation in mucins and mucin-like proteins. Fc-fused seven-peptide KEPAPTT and octapeptide KEPAPTAT recombinant proteins migrated faster than other recombinant octapeptides (Fig. 3B) and were not detected by all mAb’s (Fig. 3). This led us to hypothesize that KEPAPTT and KEPAPTAT were not glycosylated and that O-linked glycosylation (Fig. 3B) is required for recognition by several mAbs. We tested this by enzymatically digesting O-linked oligosaccharides from purified human lubricin and recombinant protein (Fig. 3A). Removal of oligosaccharides reduced lubricin’s molecular weight as demonstrated by immunoblotting (data not shown) with a polyclonal anti-lubricin antibody J108N that detects a polypeptide motif in the protein’s amino-terminal domain (14). Digestion with neuraminidase and O-glycosidase abolished immunoreactivity for mAb-7h12 and mAb-5c11, and reduced mAb-9g3 and mAb-6a8 immunoreactivity (Fig. 3A). Interestingly, T-Fc digested by neuraminidase alone had an increased immunoreactivity to mAb-7h12, mAb-5c11, and mAb-6a8 (Fig. 3A), and purified lubricin digested with neuraminidase also showed this increase (data not shown), suggesting sialic acid residues decrease or block these mAbs’ epitope. Bacterially expressed T-Fc and Mu1 were not immunodetectable with mAb-7h12 (data not shown), which is also consistent with this and other antibodies detecting a combined peptide and O-linked glycosylated epitope. Changes in gel mobility of the glycosidase digested recombinant O-linked epitope in these experiments can be attributed to sialic acid removal (Fig. 3C).

### A competition ELISA using mAb-7h12 measures lubricin in human serum, plasma, and synovial fluid

**Fig 4:**
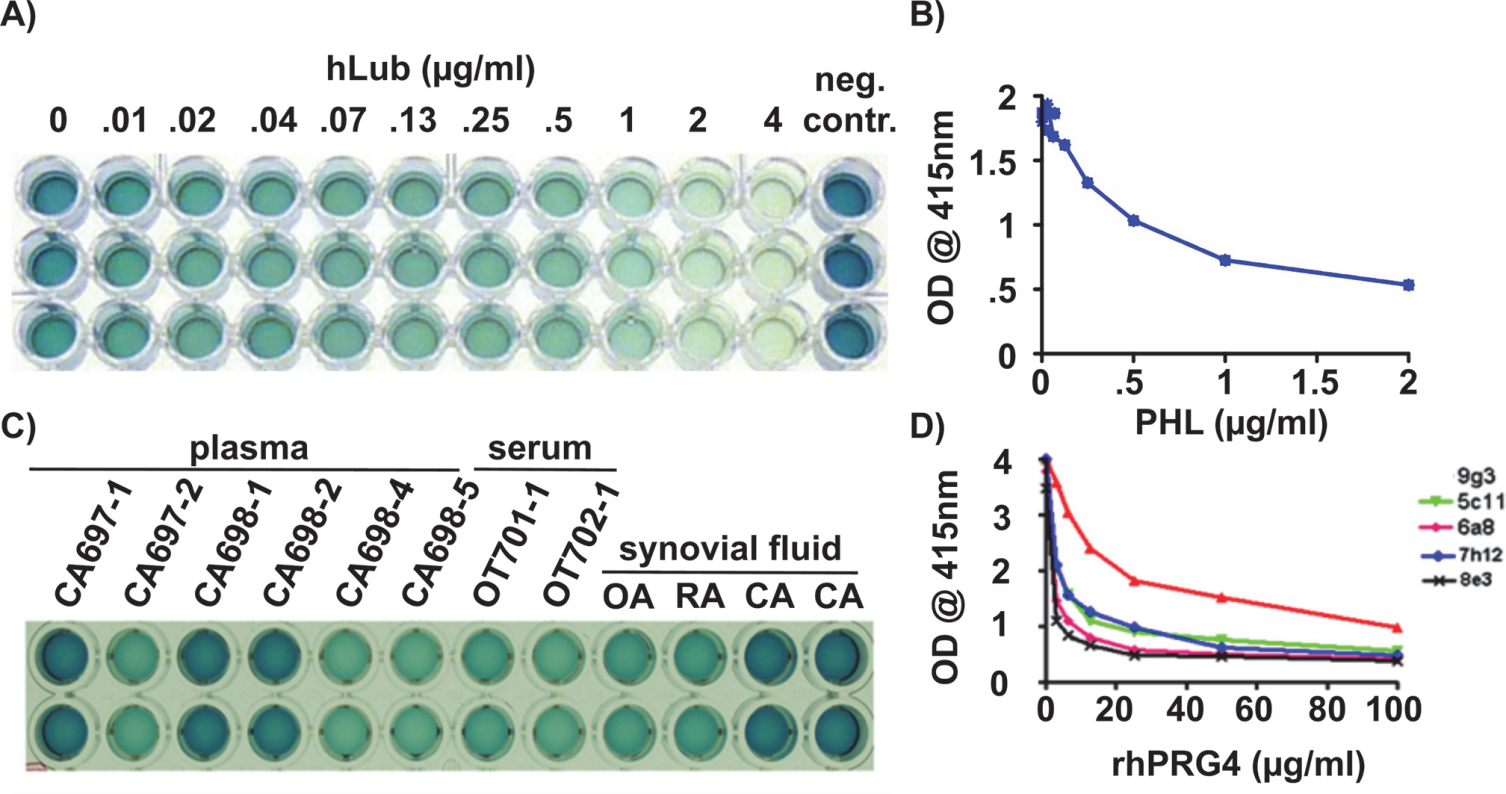
mAb-7h12 can measure lubricin in human plasma, serum, and synovial fluid by competition ELISA. **(A)** Photograph showing the result of a competition ELISA performed in triplicate. Biotin-labeled mAb-7h12 (0.2 μg/ml) was pre-incubated with purified lubricin (the concentrations of purified lubricin are indicated on top of each column) and added to lubricin-coated wells. After several washes, the mAb bound to the lubricin-coated wells, was detected colorimetrically using horseradish peroxidase (HRP) conjugated to streptavidin. Note when antibody is not pre-incubated with lubricin (0 μg/ml), all antibody binds to the pre-coated wells and the HRP-streptavidin detection of bound antibody turns these wells dark blue. In contrast, pre-incubating the antibody with increasing amounts of purified lubricin reduces antibody binding to the wells and there is less color formation by HRP-streptavidin. **(B)** Standard curve derived from the O.D. 415 nm values for the competition ELISA shown in panel **A**. The x-axis indicates the concentration of lubricin (μg/ml) that was pre-incubated with the antibody; the y-axis indicates the average difference in O.D. reading compared to the 0 μg/ml lubricin control. **(C)** Photograph showing the result of a competition ELISA performed in duplicate (individual rows). Biotin-labeled mAb-7h12 (0.2 μg/ml) was pre-incubated with 1:50 dilutions of human plasma or serum, or 1:50,000 dilutions of human synovial fluid. The plasma samples are from patients with CACP (CA697–1, CA698–1, CA698–2) and their unaffected family members (CA697–2, CA698–4, CA698–5). The serum samples are from healthy controls (OT701–1; OT702–1). The synovial fluid samples are from patients with OA, RA, or CACP (CA). Note that plasma and synovial fluid from patients with CACP do not reduce antibody binding to lubricin-coated wells as indicated by the dark blue color, whereas plasma, serum, or synovial fluid from unaffected individuals does reduce antibody binding to the lubricin-coated wells. Based upon the measured O.D. values for these samples (data not shown), no detectable lubricin is present in serum and synovial fluid from patients with CACP, whereas ~ 200 μg/ml is present in the RA and OA synovial fluid samples and ~ 0.2 μg/ml is present in the plasma and serum samples from unaffected relatives and controls. **(D)** mAbs 9g3, 5cll, 6a8 and 7h12 can be used interchangeably with recombinant human lubricin in a competition ELISA format.

To determine whether lubricin in other body tissues originates predominantly from synovial joints, we developed a competition ELISA and measured lubricin epitopes in urine, serum, and plasma. We validated the competition ELISA using known amounts of purified human lubricin (Fig. 4). As expected, there was an inverse correlation between the amount of lubricin that was pre-incubated with mAb-7h12 and the degree of antibody binding to lubricin-coated ELISA wells (Fig. 4A). A reproducible correlation occurred when the concentration of lubricin that was pre-incubated with antibody ranged from 0–2 μg/ml (Fig. 4B). When testing biologic specimens that contain lubricin as a positive control we used dilutions of synovial fluid from patients with OA and RA. As a negative control, we used dilutions of synovial fluid from patients with CACP (Fig. 4C). The competition ELISA could measure lubricin in 1:500 dilutions of RA and OA synovial fluid and did not detect lubricin in CACP synovial fluid. The competition ELISA could also measure lubricin in 1:50 dilutions of serum and plasma (Fig. 4C), but not in urine (data not shown). As expected, plasma from individuals with CACP (CA697–1, CA698–1, CA698–2) lacked lubricin, while plasma and serum from unaffected relatives (CA697–2, CA698–4, CA698–5) and unrelated controls (OT701–1, OT702–1) had quantifiable amounts of lubricin.

There was also an inverse and reproducible correlation between the amount of rhPRG4 that was preincubated with each of the individual mAbs and the degree of antibody binding to a rhPRG4 coated ELISA well (Fig. 4D). For most mAbs a quasi-linear correlation was observed when the concentration of rhPRG4 pre-incubated with antibody ranged from 0–2 μg/ml; mAb-9g3 had a quasilinear correlation when rhPRG4 ranged from 0–20 μg/ml.

Having found lubricin epitopes in serum and plasma, we next examined their characteristics. Specifically, we wanted to know whether they were within an intact or degraded mucin-like domain. After separating serum proteins by size exclusion, the immunodetectable epitopes remained in the large molecular weight (>100 kDa) fraction, suggesting that the mucin-like domain in serum is intact (data not shown). We obtained the same result when synovial fluid proteins were similarly fractionated (data not shown). Taken together, these results suggest that the immunodetectable lubricin in serum does not represent degradation products from synovial fluid. Further support for this conclusion derives from the comparison of serum lubricin levels in 30 patients with active RA and their age/sex-matched controls (Table 1), which found no significant difference.

**Table 1:** Serum lubricin levels in 30 individuals with active rheumatoid arthritis (RA) and 30 age- and sex- matched controls (Con).

RA ID	Sex	Age	CRP	Lub (ng/μl)	Con ID	Sex	Age	Lub (ng/μl)
00500	F	59	29.7	1.08	DL0291	F	59	1.06
00264	F	55	31.4	1.00	DL0026	F	55	0.65
00809	M	68	33.6	0.97	DL0440	M	68	0.79
00867	M	53	33.7	1.02	DL0014	M	53	0.86
00594	M	58	33.7	1.20	DL0024	M	58	0.97
00490	F	69	35.2	0.79	DL0067	F	69	0.74
00231	M	58	37.2	0.92	DL0090	M	58	1.02
00481	M	57	37.2	0.90	DL0227	M	57	1.15
00769	F	48	37.3	1.20	DL0074	F	48	0.64
00606	F	60	37.9	1.20	DL0160	F	60	1.29
00813	F	50	39.0	1.03	DL0071	F	50	1.00
00443	M	48	39.9	0.90	DL0012	M	48	0.90
00282	M	63	41.0	0.99	DL0017	M	63	0.70
00852	F	72	41.9	0.97	DL0664	F	73	1.24
00819	F	44	43.7	0.79	DL0101	F	44	1.00
00513	F	54	45.6	1.41	DL0202	F	54	0.70
00156	F	44	50.2	0.96	DL0129	F	44	1.20
00779	F	53	53.2	1.21	DL0128	F	53	0.67
00519	F	68	57.3	1.30	DL0773	F	68	0.95
00152	F	49	61.0	0.98	DL0295	F	49	0.85
00261	M	46	61.8	0.63	DL0056	M	46	0.86
00854	F	65	64.1	0.90	DL0033	F	65	0.95
00634	F	71	74.1	0.89	DL0739	F	70	0.73
00512	M	48	76.9	0.76	DL0057	M	48	0.54
00536	F	38	88.3	0.93	DL0413	F	38	0.82
00232	M	70	90.4	0.53	DL0016	M	70	0.72
00645	M	65	90.9	0.98	DL0032	M	65	0.95
00777	F	47	109	0.75	DL0047	F	47	0.82
00615	F	79	113	1.14	DL0692	F	79	0.82
00506	M	68	145	0.88	DL0479	M	68	1.03

The Pearson correlation coefficients between serum lubricin level and age were both 0.08 in cases and controls. The correlation between serum lubricin level and C-reactive protein level in cases was -0.31. No significant difference in mean serum lubricin levels between cases and controls was observed using a two-tailed, paired, student t-test (*p* = 0.07).

**Fig 5:**
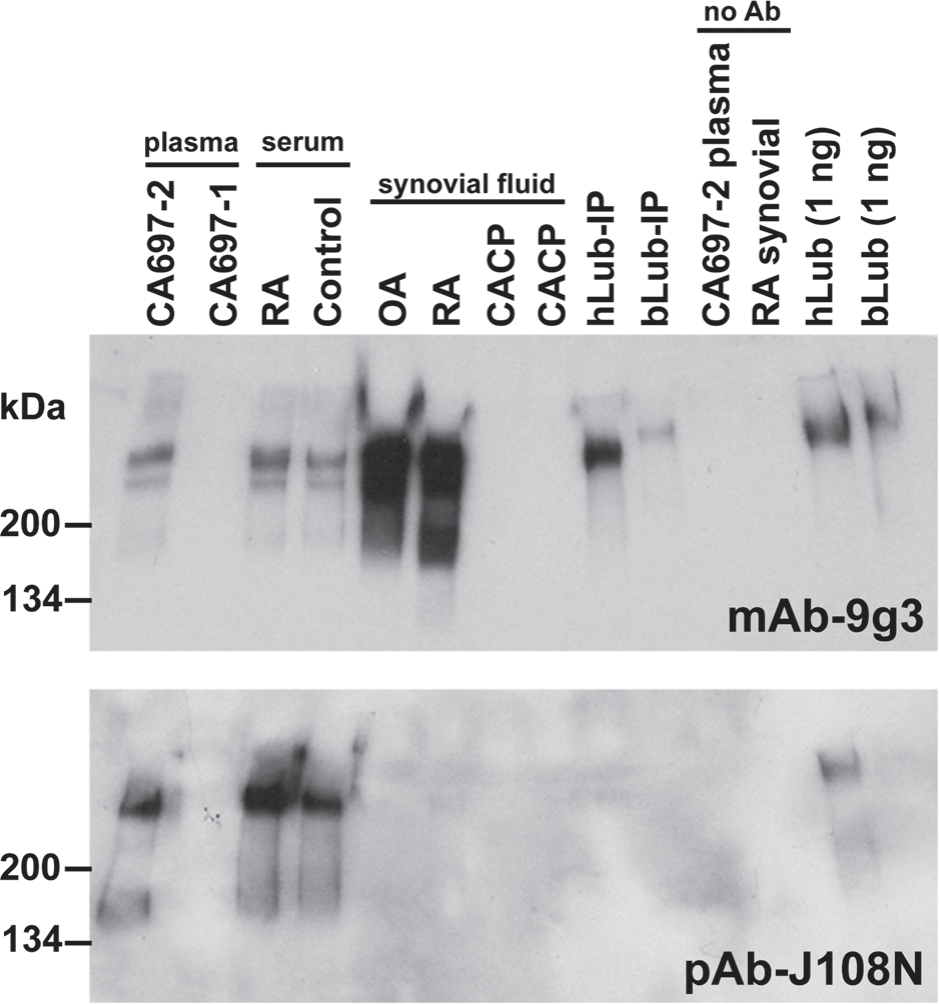
Immunoprecipitation of lubricin from human serum, plasma and synovial fluid. Biotin-labeled mAb-7h12 was mixed with plasma samples from a patient with CACP (CA697–1) or his unaffected father (CA697–2), serum samples from a patient with rheumatoid arthritis (RA) or an age- and sex-matched healthy control (Control), synovial fluid samples from a patient with osteoarthritis (OA), rheumatoid arthritis (RA), or CACP (CA), and with human (hLub-IP) and bovine (bLub-IP) lubricin that had been purified from synovial fluid. Antibody was recovered using streptavidin beads and proteins co-precipitated with mAb-7h12 were eluted, subjected to SDS-PAGE, transferred to PVDF, and immunodetected with mAb-7h12 (upper panel) or polyclonal Ab-J108N (lower panel). Note mAb-7h12 immunoprecipitated lubricin from plasma and serum that is also recognized by polyclonal antibody J108N. In contrast, lubricin immunoprecipitated from OA and RA synovial fluid is not recognized by polyclonal antibody J108N. J108N also does not recognize immunoprecipitated purified human lubricin (hLub-IP). The is no non-specific binding of lubricin to streptavidin beads as demonstrated by the lack of immunodetectable protein when mAb-7h12 is not used in the co-IP (no Ab control) of plasma from the unaffected CACP parent or synovial fluid from a patient with RA. As positive controls for immunodetection purified human and bovine lubricin (hLub and bLub) were directly subjected to SDS-PAGE for immunodetection.

We also immunoprecipitated (IP) lubricin from blood and synovial fluid with mAb-7h12 and then probed the precipitated protein with mAb-7h12 and polyclonal Ab-J108N; the latter polyclonal antibody detects an epitope within the amino-terminal domain of all *PRG4* spliceforms [23]. As expected, no lubricin protein could be immunoprecipitated from plasma or synovial fluid from patients with CACP (Fig. 5). If lubricin in blood originated in the synovial fluid and represented a degradation product, we expected it to lack the amino-terminal domain. Surprisingly, we obtained the opposite result. That is, the fraction of lubricin molecules having an intact amino-terminal domain epitope is larger in blood than in synovial fluid (Fig. 5).

### Lubricin is immunodetected in healthy synovium and synovium from patients with acquired joint disease, but not in synovium from patients with CACP

**Fig 6:**
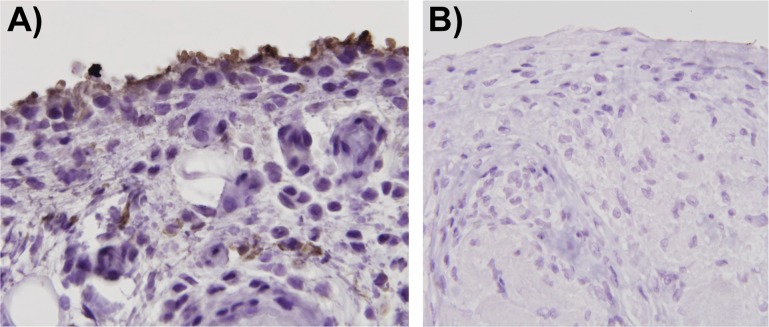
Immunodetection of lubricin in synovial tissue. Photomicrographs of tissue sections from archival human synovial biopsies from patients with osteoarthritis **(A)** or CACP **(B)**. Sections had been incubated with mAb-7h12 and detected with avidin/biotin/peroxidase. Note immunodetectable lubricin (brown-colored stain) is concentrated along the surface of OA synovium, and not found in CACP synovium. The OA sample was counterstained with hematoxylin & eosin and imaged at 1000X magnification. The CACP sample was counterstained with eosin and imaged at 600X magnification.

We performed immunohistochemistry using mAb-7h12 on paraffin embedded formalin fixed synovial tissue sections from patients with osteoarthritis and pigmented villonodular synovitis and on synovium, obtained at autopsy, from an individual without joint disease. In addition, we studied synovial biopsy samples from two unrelated patients with CACP. In contrast to finding immunodetectable lubricin in the synovium of a healthy joint or a joint with an acquired disease, we did not detect lubricin in synovium from a CACP joint (Fig. 6).

## Discussion

The precocious joint failure that occurs in patients with CACP, a genetic deficiency of lubricin, reveals the importance of this protein for protecting joints [12]. Acquired deficiency of lubricin, as can occur following trauma, infection, or inflammation may also hasten the onset or progression of joint failure, where loss of chondrocytes appears to be linked to excessive friction at the joint surface [18]. Although lubricin is an abundant protein in synovial fluid, its synthesis, post-translational modification, localization, and degradation remain incompletely understood. We have generated and characterized sensitive and specific mAbs against human lubricin to further our understanding of these processes.

These antibodies detect a combined oligosaccharide/peptide epitope that occurs multiple times within the protein’s first mucin-like domain. The presence of multiple copies of this epitope accounts for the antibodies’ sensitivity, while the uniqueness of the epitope sequence explains the antibodies’ specificity. Interestingly, O-linked oligosaccharides are part of the epitope, yet the epitope may be partially blocked when these oligosaccharides are capped by sialic acid. The mAbs reported here detected lubricin in synovial fluid from multiple species, but did so with differing sensitivity. This may be due to differences in the number of KEPAPTTT repeats (e.g., humans have 26 repeats, mice have 7), the nature of the linked oligosaccharides, or the extent to which they are sialylated. Since glycosylation is essential to the protein’s lubricating function [20], it may become valuable to employ antibodies, such as mAb-7h12 and mAb-5c11, to determine whether the extent and nature of glycosylation change during aging or as a consequence of disease [21].

The mAbs could detect lubricin in serum and plasma by competition ELISA. Previously, we demonstrated absence of lubricin in synovial fluid from patients with CACP [23]. Here we show that lubricin was undetectable in blood from several patients with CACP, but detectable in their unaffected relatives and controls. Since most identified CACP disease-causing mutations in *PRG4* abolish lubricin production, the sensitivity of the competition ELISA as a diagnostic test for CACP should be high; however, other diagnostic strategies including sequencing of *PRG4*, will be required for the minority of patients with CACP who have mutations that affect lubricin function rather than production.

Because the epitopes of the mAbs were within the protein’s mucin domain we have been able to gain insight into the mechanism by which lubricin is degraded within joints. In contrast the gastrointestinal and respiratory tracts, where mucins are degraded by glycosidases and peptidases expressed by resident bacteria, synovial joints are sterile thereby precluding this mechanism of degradation. We used a competition ELISA to compare the abundance of lubricin epitopes in the serum of patients with active inflammatory joint disease to age- and sex-matched controls and we found no difference. This suggests that the glycosylated KEPAPTTT epitope in serum does not originate from synovial joints. Although this result does not preclude other lubricin fragments in serum from originating in synovial fluid, it implies that degradation of the mucin-like domain occurs intracellularly with little or no release of glycosylated oligopeptide. Further support for this conclusion derives from the failure to find small molecular weight fragments from the mucin-like domain in synovial fluid.

Even though the mucin-like domain of lubricin is degraded intracellularly, it is clear that other lubricin domains are subjected to extracellular degradation. For example, the polyclonal Ab-J108N, detected full-length lubricin and degradation fragments containing the protein’s amino-terminal globular domain in synovial fluid from patients with active RA [23]. Therefore, comparing ratios of intact and degraded lubricin within synovial fluid may be useful in monitoring disease status. Interestingly, most lubricin molecules within synovial fluid do not contain the epitope recognized by J108N, which differs from what we observed for blood. Whether this difference represents a post-translational modification of the amino-terminal domain that hides the epitope or cleavage of this domain from the remainder of the protein remains to be determined.

Other antibodies have previously provided insight into lubricin biology. Monoclonal antibodies (3-A-4 and 6-A-1) generated against the carboxyl-terminal globular domain of bovine lubricin demonstrated lubricin expression by superficial zone chondrocytes and synoviocytes [27], localization at cartilage [28] and meniscal [29] surfaces, and the compressive surfaces of tendon [30]. Antibody 3-A-4 was also used to demonstrate the ability of lubricin in synovial fluid to re-adhere to a cartilage surface that had been stripped of lubricin with 1.5 M NaCl [31]. However, neither antibody is able to immunodetect human lubricin. A polyclonal antibody, 06A10, generated against an unspecified truncated form of recombinant human lubricin was used immunohistochemically to detect sheep lubricin [32]. This antibody was also used to show that recombinant human lubricin, which had been reduced and alkylated, did not avidly bind to the cartilage surface compared to non-reduced protein [32]. Several monoclonal antibodies generated against lubricin (SZP) recovered from cultured human chondrocytes have been used for immunohistochemistry, western blotting, and sandwich ELISA [33]. Although the epitopes recognized by these antibodies were not reported, a surprising observation was the ability of one monoclonal antibody (S13.52) to recognize lubricin purified from human synovial fluid but not from articular cartilage. Of even greater surprise was that this antibody detected similar abundances for lubricin in serum and synovial fluid. This result is surprising since we estimated the abundance of lubricin in serum at 0.2 μg/ml and in synovial fluid at 200 μg/ml using mAb-7h12 and the competition ELISA (Fig. 4). Our quantification may not accurately reflect protein abundance if the extent or type of lubricin O-linked glycosylation found in serum differs from that found in synovial fluid. However, the studies that employed S13.52 and S6.89 did not utilize serum and synovial fluid from patients with CACP as negative controls, so cross reactivity with another serum protein may explain their result. Monoclonal antibody S6.89 has been used in a number of other species recognizing cow, rabbit, guinea pig [33], and dog [34]. Of the present monoclonal antibodies we report here, mAb-9g3 has been used immunohistochemically in rats [35] and mAb-5c11 in cow [6].

In conclusion, we have produced a set of sensitive mAbs that are specific to a repetitive, glycosylated, octapeptide motif of human lubricin, and used one of them to improve the laboratory diagnosis of a Mendelian genetic skeletal disease CACP, to distinguish degradation of lubricin that occurs intracellularly from that which occurs extracellularly, and to begin delineating how monitoring the extracellular degradation of lubricin can be used to assess joint health. Investigators studying other animal models of joint disease may find these mAbs useful, since they detect lubricin from several species.
